# Carbon stocks of three secondary coniferous forests along an altitudinal gradient on Loess Plateau in inland China

**DOI:** 10.1371/journal.pone.0196927

**Published:** 2018-05-03

**Authors:** Ning Liu, Hongwei Nan

**Affiliations:** School of Forestry, Shanxi Agricultural University, Taigu, Shanxi Province, P. R. China; Pacific Northwest National Laboratory, UNITED STATES

## Abstract

Natural forests in inland China are generally distributed in montane area and secondary due to a semi-arid climate and past anthropogenic disturbances. However, quantification of carbon (C) stock in these forests and the role of altitude in determining C storage and its partition among ecosystem components are unclear. We sampled 54 stands of three secondary coniferous forests (*Larix principis-rupprechtii* (LP) forest, *Picea meyerii* (PM) forest and *Pinus tabulaeformis* (PT) forest) on Loess Plateau in an altitudinal range of 1200-2700m a.s.l. C stocks of tree layer, shrub layer, herb layer, coarse wood debris, forest floor and soil were estimated. We found these forests had relatively high total C stocks. Driven by both higher vegetation and soil C stocks, total C stocks of LP and PM forests in the high altitudinal range were 375.0 and 368.4 t C ha^-1^ respectively, significantly higher than that of PT forest in the low altitudinal range (230.2 t C ha^-1^). In addition, understory shrubs accounted for about 20% of total biomass in PT forest. The proportions of vegetation to total C stock were similar among in the three forests (below 45%), so were the proportions of soil C stock (over 54%). Necromass C stocks were also similar among these forests, but their proportions to total C stock were significantly lower in LP and PM forests (1.4% and 1.6%) than in PT forest (3.0%). Across forest types, vegetation biomass and soil C stock simultaneously increased with increasing altitude, causing fairly unchanged C partitioning among ecosystem components along the altitudinal gradient. Soil C stock also increased with altitude in LP and PT forests. Forest floor necromass decreased with increasing altitude across the three forests. Our results suggest the important role of the altitudinal gradient in C sequestration and floor necromass of these three forests in terms of alleviated water conditions and in soil C storage of LP and PM forests in terms of temperature change.

## Introduction

Montane forests are forests distributed at mid and low altitudes and a key component of mountain ecosystems [1]. Montane forests are generally fragmented and in secondary growth due to intense anthropogenic disturbances[1–3]. Even so, high growth temperature in montane area and high productivity of dominating pioneer species after disturbances can benefit carbon (C) sequestration and storage of these forests, making them a potential C sink for the purpose of mitigating the effect of climate warming[4–5]. However, less attention was paid to montane forests for their C stocks and the ecological factors controlling C storage and its partition among ecosystem components, especially to those that are distributed on Loess Plateau in China.

The Loess Plateau in north-central China is the cradle of Chinese civilization. Long history of intense human activities and a semi-arid monsoon climate had made the Loess Plateau an ecologically fragile region with less than 10% of forest coverage, mostly on mountains in an altitudinal range of 1200-3000m a.s.l. [3]. These montane forests, dominated by pioneer conifers and broadleaves, are generally protected in the past several decades for their ecological services and thus becoming a potential major C sink in the region. However, previous studies on forest C budget on Loess Plateau were mainly conducted in young stands and artificial plantations, while the ecological drivers of C stock were seldom explored[6–9]. As the characterized low precipitation and high vapor pressure deficit (VPD) on the Loess Plateau would make these forests more susceptible to the climate change[10], it is also necessary to determine the ecological drivers of forest C stock to predict the response of C balance to the future climate. In a forest inventory study carried out on Loess Plateau, Zhang et al. [11] found a positive relationship between elevation and forest C density based on the volume-derived biomass of live trees, indicating the potential influence of the altitude in determining forest C stock in this region, while the effect of altitude on total C storage and its partition among all ecosystem components still remains unanswered.

The distribution and performance of plants on mountains are affected by changing environmental factors with altitude. Among these factors, the changes of temperature and precipitation are most significant, thus providing excellent test grounds for predicting the responses of plants, communities and ecosystems to the climate change[12]. Studies on variations of forest C stock along altitudinal gradients were fewer than others, such as plant ecophysiology, biodiversity and tree line migration[13–16]. Despite a warm climate is in favor of plant growth, it seems that the high altitude forests may not be inferior in biomass accumulation in comparison to the low altitude ones under favorable site conditions[17–18]. Soil C pool also tends to increase with increasing altitude as soil respiration may be decreased by low temperature at high altitudes[19]. However, varied responses of C stock in different ecosystem components to altitudinal gradients were also reported across forest types[20–22]. Furthermore, in previous studies, changes of forest aboveground and soil C stocks along altitudinal gradients were often accompanied with transition of forest types[19, 22]. Since many tree species can grow in a wide range of altitudes, altitudinal variations of forest C stock should also be examined under similar tree species composition in supplement to our understanding of C balance in forest ecosystems under the future climate.

The objective of this study is to examine the carbon content and its distribution among ecosystem components along an altitudinal gradient in three secondary coniferous forests on the Loess Plateau dominated by *Larix principis-rupprechtii* Mayr. (Prince Rupprecht’s larch), *Picea meyrii* Rehd. et Wils. (Meyer spruce) and *Pinus tabulaeformis* Carrière (Chinese pine) respectively. These three forests were chosen because coniferous forests dominate the landscape on the Loess Plateau in a relatively continuous coverage and are better preserved than broadleaved forests, accounting for about half of the total forested land with a total area of 32,900 km^2^ in total, of which these three forests account for more than 90% collectively, growing at various altitudes under different climate conditions[3]. The altitudinal gradient was used as a surrogate to climate warming in this study, especially for concurrent changes of temperature and water conditions. Specifically, we hypothesized that in these three forests, C stock is generally high after decades of forest regrowth and the altitudinal gradient plays an important role in determining forest C stock depending on forest type. To test our hypotheses, we 1) quantified the C stock in these three forests by ecosystem components, i.e. vegetation, necromass and soil, to compare with each other and with other forests and regional and national averages; and 2) examined the effect of altitude on total C storage and its partition among ecosystem components across and within forest types by simple regressions.

## Materials and methods

### Study area

**Fig 1:**
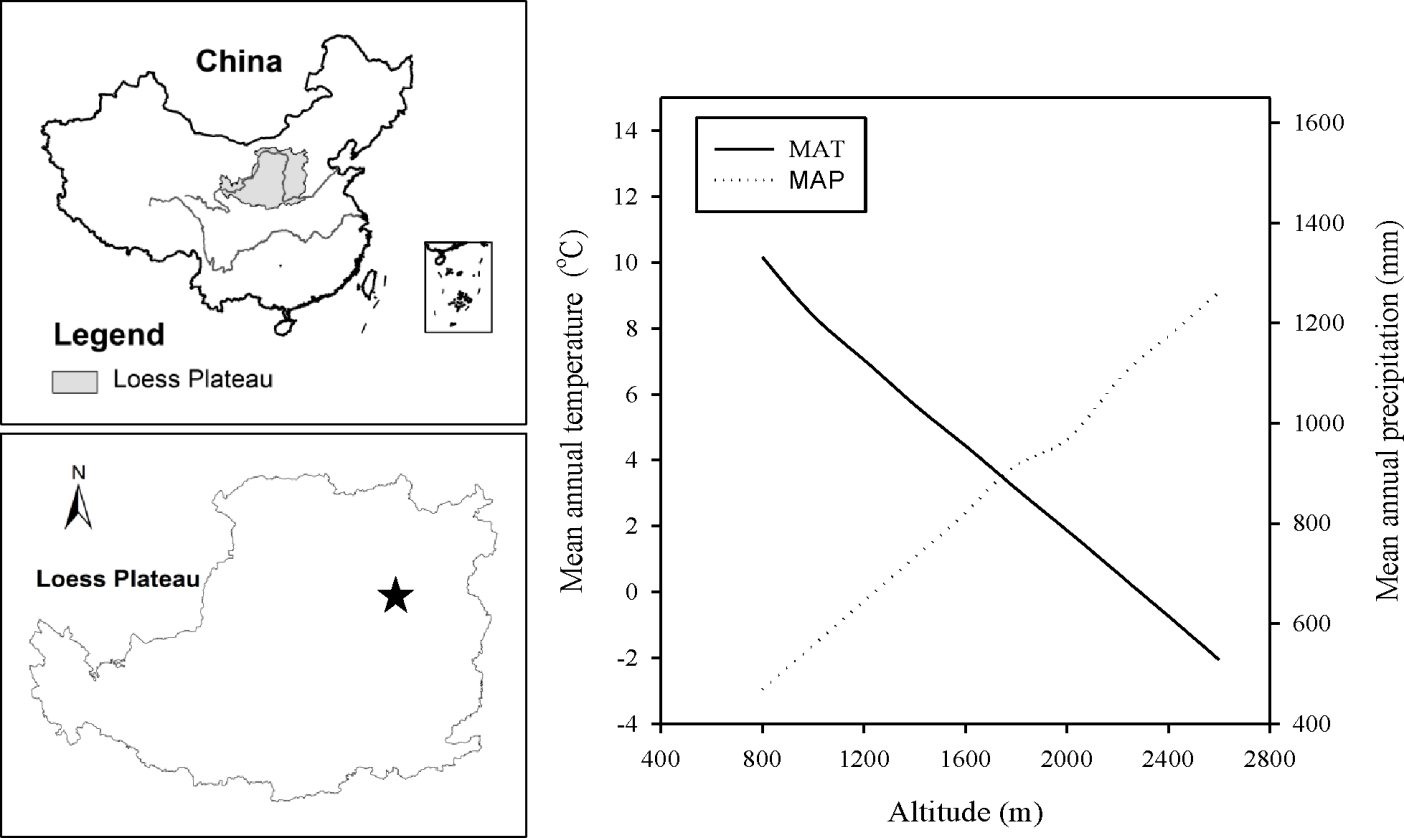
Schematic maps showing the location of the study area on the Loess Plateau, China and the mean annual temperature and precipitation changes along the altitudinal gradient on Guandi mountain range. MAT, mean annual temperature; MAP, mean annual precipitation. MATs and MAPs along the altitudinal gradient are presented using the estimated data given by Xiao et al.[23].

The study was conducted on the Guandi Mountain Range (111°41′-112°08′ E and 37°45′-37°94′ N) at the eastern edge of the Loess Plateau, Shanxi Province, China (Fig 1). The area of forested land in the study area is about 276,596 ha, over half of which are secondary forests. The forested land is managed by Guandi Mountain State Forest Administration Bureau which grants access to the School of Forestry, Shanxi Agricultural University for forest researches and field trips. The study area is located in temperate semi-arid zone with a continental monsoon climate. The mean annual temperature (MAT) and the mean annual precipitation (MAP) for past 30 years are about 7°C and 650mm respectively at the nearest meteorological station 30km west to the study area. The annual evapotranspiration is estimated over 900mm in this region. Estimated with the climate model for the study area developed by Xiao et al.[23], the MAT increases linearly with a rate of 0.66°C per 100m as the elevation decreases from 2600m to 800m a.s.l. in the study area, while the MAP decreases linearly with a rate of 43.85mm per 100m (Fig 1). The soil parent material in the forested area is aeolian loess with a depth from 1m to 30m. The forest soil is classified as mountain brown soil series. Humus horizon is shallow and varies from 0 cm to 4 cm in depth depending on stand type and age.

The study area was intentionally burned periodically to allow grass grow for herding till 1900s and left to natural regeneration of trees thereafter. From 1950s to 1980s, timber harvesting cleared much of these secondary forests except those were protected. In 1980s, commercial logging was prohibited by Chinese government, while continuous fuel collecting activities of local people nearly destroyed all broadleaved stands. Thus, naturally regenerated coniferous forests dominate the landscape in the study area today.

In the study area, intolerant Prince Rupprecht’s larch and tolerant Meyer spruce often form pure and mixed stands in the high altitudinal range from 1600m to 2800m a.s.l., and Chinese pine is the only dominate conifer in the low altitudinal range from 1000m to 1800m a.s.l.[24]. Meanwhile, several broadleaved species, e.g. early successional *Populus cathayana* Rehd. (Cathay poplar) and *Betula platyphylla* Suk. (Asian white birch), and late successional *Quercus liaotungensis* Mayr. (East-liaoning oak), *Acer ginnala* Maxim. (crimsonleaved maple) and *Sorbus pohuashanensis* Mayr. (mountain ash), are also present in the study area, mostly mixing with conifers or forming scattered pure stands[25].

### Sampling design

A total of 54 stands were selected in this study. For each of three conifer stand types, i.e. Prince Rupprecht’s larch (LP), Meyer spruce (PM) and Chinese pine (PT) forests, 18 stands were randomly sampled along the altitudinal gradient. These stands are all naturally regenerated and fully stocked (with a canopy closure over 80%). The selected stands are at least 1 ha in area and located on shade and half-shade slopes (east-, northeast-, north- and northwest-facing slopes) at mid slope. Because the distribution of forests on the Loess Plateau is primarily limited by water, forested stands on sun slopes and mountain tops are usually understocked in the study area, thus were not sampled. Down-slope stands were not sampled too since the rooting layer in these stands is mainly filled with coarse sands and gravels instead of soil materials. Furthermore, all selected stands were visually confirmed to have no severe human disturbances by checking the presence of logging stumps. For each stand, the basal area of dominate conifer species exceeded 75% of total basal area. Number of stands and stand characteristics were presented in Table 1A.

**Table 1:** Stand characteristics and vegetation biomass and its components by forest type in three coniferous forests on the Loess Plateau, China.

	LP	PM	PT	One-way ANOVA *p* values
a) Stand characteristics				
Elevation (m)	2162(64)a	2113(60.6)a	1585(41.8)b	<0.001
Slope (degrees)	16.9(2.1)a	15.7(2.4)a	21.11.8)a	0.186
Stand age (yrs)	63(2.5)a	61(2.3)a	66(3.3)a	0.407
Density (stem ha^-1^)	781(75.8)ab	981(90.6)a	620(55.3)b	0.006
Basal area (m^2^ ha^-1^)	43.5(2.5)ab	45.5(2.6)a	34.2(4.2)b	0.037
Percentage of dominant conifer (%)	89.3(2.3)	90.5(2.5)	94.2(1.6)	
b) Biomass				
Vegetation (t ha^-1^)	276.0(16.0)a	316.6(19.2)a	206.8(21.6)b	<0.001
Tree layer (t ha^-1^)	260.7(17.5)a	310.3(19.3)a	170.1(23.3)b	<0.001
Shrub layer (t ha^-1^)	14.8(4.3)b	5.8(1.3)b	36.4(4.5)a	<0.001
Herb layer (t ha^-1^)	0.53(0.07)a	0.50(0.05)a	0.33(0.04)b	0.015
MAI (t ha^-1^ yr^-1^)	4.4(0.2)a	5.2(0.3)a	3.1(0.2)b	<0.001

Note: Data are presented by plot means with 1 standard error in parentheses. There are 18 plots for each forest type. Different letters within same row indicate significant differences (p<0.05) among three stand types. LP, *Larix principis-rupprechtii* forest; PM, *Picea meyerii* forest; PT, *Pinus tabulaeformis* forest.

In summer 2015, a 20 × 20 m (0.04 ha) sample plot was established at the center of each selected stand. All living trees reaching breast height within each plot were identified for species and measured for diameter at 1.3m height (DBH). Tree height was estimated using Chapman–Richards function[26]. Heights (H) of 24–30 trees per species randomly selected at all stands were measured by a laser rangefinder to fit against DBH with following model:

$$H=1.3+a{\left(1-e^{-bDBH}\right)}^{c},$$ (1)

where *a*, *b*, and *c* are regression coefficients. Fitted DBH-H models were presented in S1 Table. Tree saplings (less than 1.3m in height) and shrubs were also identified and measured for height and root collar diameter (RCD). One to three trees of dominant conifer species in different diameter classes were selected and cored at 1.3m in each plot to develop species-specific DBH-age models as follows:

$$Age=a+bDBH^{c},$$ (2)

where *a* is the estimated age when a certain species reaches 1.3m in height based on experience, and *b* and *c* are regression coefficients. Fitted DBH-Age models were presented in S2 Table. Stand age was then calculated as the average age of all trees with a DBH ≥10cm as a conservative estimate.

All plants in a plot were categorized into three layers. Trees and shrubs were sorted into the tree layer (DBH ≥10cm) and the shrub layer (including small trees with a DBH <10cm and shrubs) based on diameter distribution; tree seedlings and shrubs (<0.5m high) and herbs were sorted into the herb layer.

### Total biomass and carbon content

Total biomass (including above ground biomass, AGB, and belowground biomass, BGB) in the tree layer was estimated with height and/or DBH using individual tree biomass models developed by Zhang and Shangguan[27] for Prince Rupprecht’s larch; by Wang et al.[28] for Chinese pine and Cathay poplar; by Wang[29] for East-liaoning oak; and by State Forestry Administration[30] for Asian white birch and Meyer spruce. These models were all developed using naturally regenerated trees in the same region (S3 Table). Total and component biomass of other broadleaves in the tree layer were estimated using the biomass models of Cathay poplar and East-liaoning oak for early and late successional species respectively.

Total biomass in the shrub layer was calculated as the sum of small trees and shrubs. Biomass of small trees higher than 4m was estimated with same species specific biomass models above. Biomass of tree saplings with a height <4m and ≥0.5m were estimated with RCD and height using species-specific biomass models developed in this study. Briefly speaking, about 1–2 tree seedlings per species outside each plot, if present, were cut down and weighted by leaves, branches and stems. Samples of different organs were brought back to lab and dried to constant weight to calculate the ratios of dry mass to fresh weight. Logarithmic models were then developed for AGB of each tree species, as shown in S4 Table. BGB was estimated by species-specific AGB/BGB ratios[31]. Biomass of shrubs was calculated as the sum of AGB and BGB. AGB of shrubs was estimated using the allometric equations developed by Chen et al.[32] for shrub species in the region. BGB was calculated by the estimated relationship between AGB and BGB[32].

Total biomass in the herb layer was determined by destructive sampling. In each plot, five 1 × 1 m subplots were randomly set up. All herbs in a subplot, including tree seedlings and shrubs <0.5m in height, were all uprooted to measure fresh weight. Portions of the samples collected in each subplot were bagged by woody tissues and herbs, and brought back to lab to dry at 80°C to a constant weight. The ratios of dry mass to fresh weight of woody tissue and herb samples were used to calculate herb layer biomass in each subplot, and then averaged and scaled up to the plot level.

The sum of tree, shrub and herb layer biomass was used to calculate vegetation biomass in a stand. The vegetation biomass was then divided by stand age to calculate mean annual increment (MAI), as an indirect indicator of the capacity of forest carbon sequestration[33].

Carbon content of individual trees was calculated as the product of total dry mass and species-specific carbon concentration reported for conifer and broadleaved species in the region by Ma et al.[31]. Carbon content of shrubs was calculated as the product of plot-level shrub layer dry mass and a constant ratio of 0.4897[31].The sum of carbon contents of trees with a DBH ≥10cm was calculated as tree layer C stock. The sum of carbon contents of small trees (DBH <10cm) and shrubs was calculated as shrub layer C stock. Herb layer C stockwas calculated as the product of plot-level herb layer dry mass and an assumed constant ratio of 0.5.

### Coarse wood debris and forest floor necromass

All sampled plots were revisited in summer 2016 to investigate coarse wood debris (CWD) and forest floor necromass. CWD only included snags and broken branches (diameter ≥2.5cm) in the sampled stands. No fallen trees or stumps were found. Snags were identified and measured for DBH and height to calculate biomass using species-specific biomass models for stems and roots. Broken branches on the ground were collected and weighed in each plot. Forest floor materials were collected in five 1 × 1m subplots and sorted into fine wood debris (including twigs with diameter <2.5cm and cones), leaves and half decomposed materials.

Samples of broken branches and sorted forest floor materials were bagged separately and dried at 80°C to a constant weight. CWD in each plot was calculated as the sum of the estimated biomass of snags and the dry mass of broken branches (diameter ≥2.5cm). Forest floor necromass in each plot was calculated as the sum of the dry mass of broken branches (diameter <2.5cm) in the plot and plot-level dry mass of forest floor materials scaled up from sub-plot average. Carbon contents of CWD and forest floor necromass in each plot were calculated using a constant ratio of 0.5.

### Soil and total carbon stock

Soil C stock was also investigated during the revisit in summer 2016. A soil profile pit was dug at the center of each plot to 1m in depth perpendicular to the slope. Soil samples were taken at five depths (0–20, 20–40, 40–60, 60–80, and 80-100cm) for measuring soil bulk density and carbon concentration. The soil samples were air dried and sieved with 2mm mesh. Rocks (diameter ≥2mm) were picked out and measured for volume. Total carbon concentration (g C kg^-1^) of each sample was analyzed by dry combustion method using a LECOL CNS 2000 Analyzer. Soil C stock in each soil horizon was calculated with its depth, volume-adjusted bulk density and total carbon concentration. Total soil C stock was calculated as follows:

$$C_{soil}=\sum_{i=1}^{5}BD_{i}\times C_{i}\times D_{i}$$ (3)

where C_*soil*_ is the total soil C stock to 1m deep, and BD_*i*_, C_*i*_, and D_*i*_ represent the bulk density after adjusting soil volume for rocks, the total carbon concentration and the depth of *i*th soil horizon.

Total C stock of each plot was calculated as the sum of vegetation, CWD, forest floor and soil C stocks. The proportions of vegetation, necromass and soil C stocks to total C stock were also calculated for each plot.

### Statistical analysis

One-way analysis of variance (ANOVA) was employed to compare stand characteristics, total and component biomass and carbon storages among the three forests. To meet normality assumptions, square-root transformations were applied on shrub- and herb-layer biomass, and log transformations were applied on the necromass of CWD and forest floor materials and the ratio of necromass to total C stock. Tukey’s test was used in post hoc multiple comparisons. Relationships between vegetation biomass components and altitude, which was used as a proxy for concurrent changes in temperature and precipitation, were examined by simple regression analyses across the three forests and within each forest type, so as to ecosystem C stock and its components. All statistical analyses were performed using R 3.2.5[34]. All data used in the analyses were publicly available in S1 File submitted as the supporting file.

## Results

### Vegetation biomass

Vegetation biomass of LP and PM forests were 276.0±16.0 and 316.6±19.2 t ha^-1^ respectively, significantly higher than that of PT forest (206.8±21.6 t ha^-1^), mainly due to higher biomass in the tree layer in the former two forests (Table 1B). About 94.4% and 98.0% of vegetation biomass were in the tree layer for LP and PM forests, but only 82.2% for PT forest. In contrast, shrub layer biomass of PT forest (about 17.6% of vegetation biomass) was significantly higher than that of LP and PM forests (5.4% and 1.8% respectively). Although accounting for a small fraction of the total biomass, less than <0.2% for all three forests on average, herb layer biomass of LP and PM forests were also significantly higher than that of PT forest (Table 1B). Meanwhile, both LP and PM forests had significantly higher MAIs, because of their higher vegetation biomass than and similar stand ages to PT forest (Table 1B).

**Fig 2:**
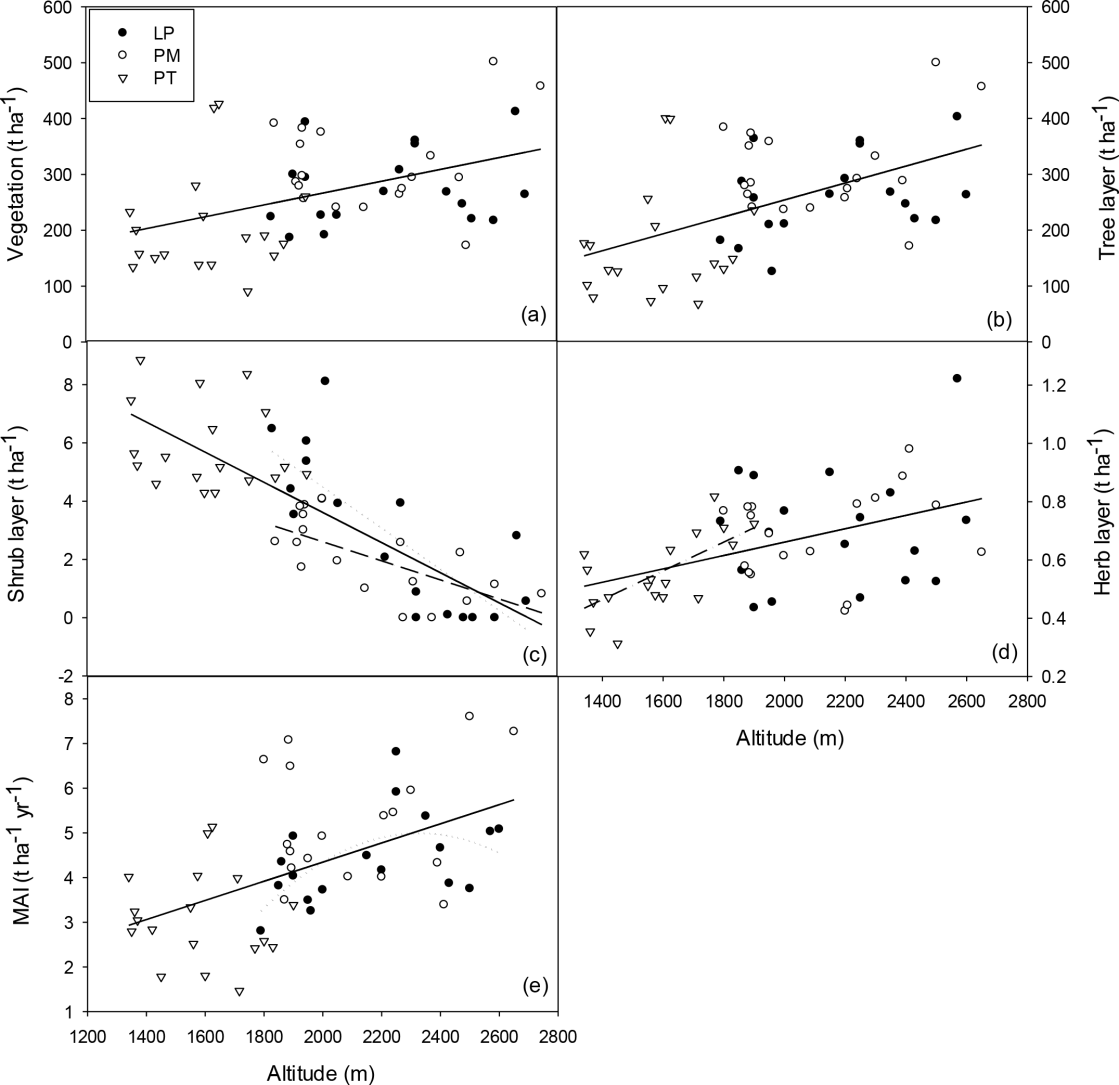
**Vegetation (a), tree layer (b), shrub layer (c) and herb layer biomass (d) and MAI (e) along the altitudinal gradient in three coniferous forests on the Loess Plateau, China.** MAI, mean annual increment; LP, *Larix principis-rupprechtii* forest; PM, *Picea meyerii* forest; PT, *Pinus tabulaeformis* forest. Shrub and herb layer biomass were presented after square-root transformation. Solid lines represent significant linear relationships between altitude and vegetation biomass (r^2^ = 0.19, p<0.001), tree layer biomass (r^2^ = 0.27, p<0.001), shrub layer biomass (r^2^ = 0.62, p<0.001) and herb layer biomass (r^2^ = 0.22, p<0.001) and MAI (r^2^ = 0.29, p<0.001) respectively across the three forests. Dotted lines represent a significant linear relationship between altitude and shrub layer biomass (r^2^ = 0.62, p<0.001) and a significant quadric relationship between altitude and MAI (r^2^ = 0.32, p = 0.057) respectively in LP forest. Long dash line represents a significant linear relationship between altitude and shrub layer biomass (r^2^ = 0.47, p = 0.002) in PM forest. Dot-dash line represents the significant linear relationship between altitude and herb layer biomass (r^2^ = 0.43, p = 0.003) in PT forest.

Across three coniferous forests, vegetation biomass and tree layer biomass both generally increased with altitude, but such relationship was not found within any of forest types in the regression analyses (Fig 2A and 2B). Shrub layer biomass decreased with increasing altitude across the three forests and within LP and PM forests respectively (Fig 2C). Similar to vegetation biomass, herb layer biomass increased with altitude across the three forests and also within PT forest (Fig 2D). Moreover, MAI showed a similar increasing pattern along the altitudinal gradient as vegetation biomass, but marginally increased with altitude curvilinearly within LP forest, reaching maximum around 2200-2400m a.s.l. (Fig 2E).

### CWD and forest floor necromass

**Fig 3:**
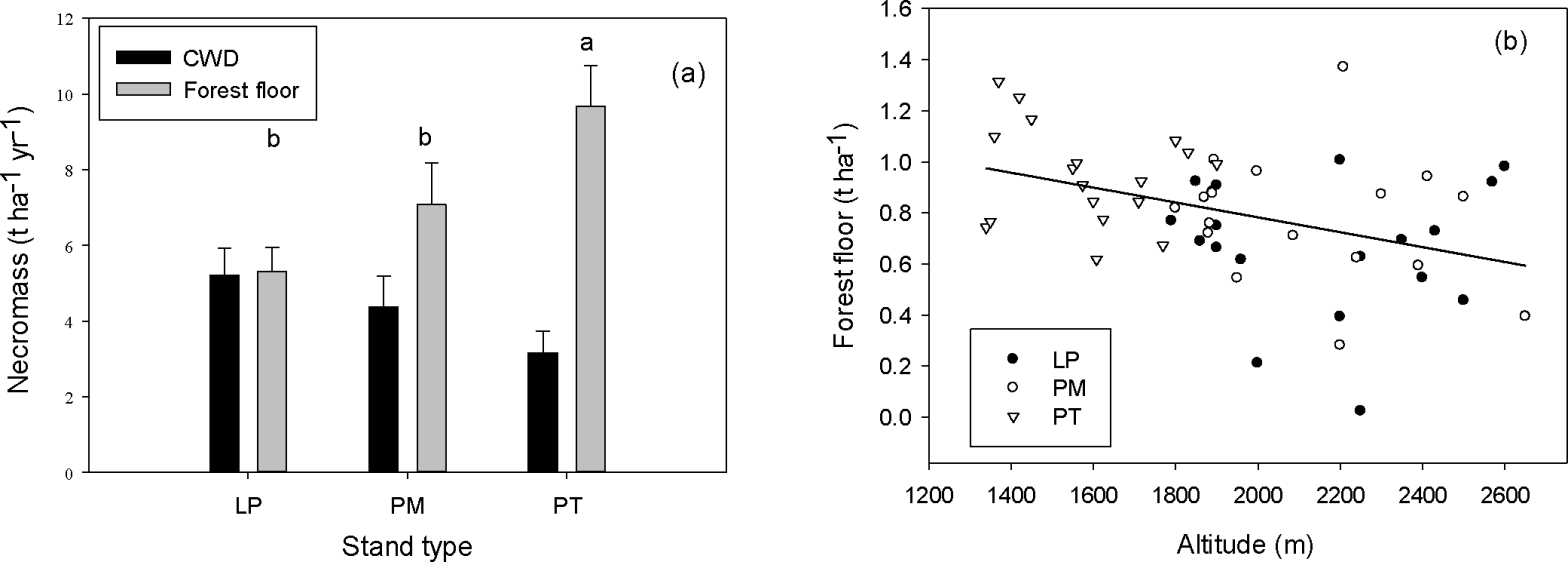
**CWD and forest floor necromass (a) and forest floor necromass (after natural-log transformation) along the altitudinal gradient (b) in three coniferous forests on the Loess Plateau, China.** LP, *Larix principis-rupprechtii* forest; PM, *Picea meyerii* forest; PT, *Pinus tabulaeformis* forest. Different letters in (a) indicate significant differences of the forest floor necromass (p<0.05) among three forest types. Solid line in (b) represents a significant linear relationship (r^2^ = 0.16, p = 0.003) between altitude and forest floor necromass across the three forests.

CWD necromass in the three forests, including snags and broken branches, were ranging from 3.2±0.6 t ha^-1^ to 5.2±0.7 t ha^-1^ and not significantly different, while forest floor necromass in PT forest (9.7±1.1 t ha^-1^) was significantly higher than that in LP and PM forests (5.3±0.6 and 7.1±1.1 t ha^-1^ respectively, p = 0.03, Fig 3A). No relationship between CWD necromass and altitude was found across or within forest types, but forest floor necromass decreased significantly with increasing altitude across the three forests (Fig 3B).

### Soil carbon stock

**Fig 4:**
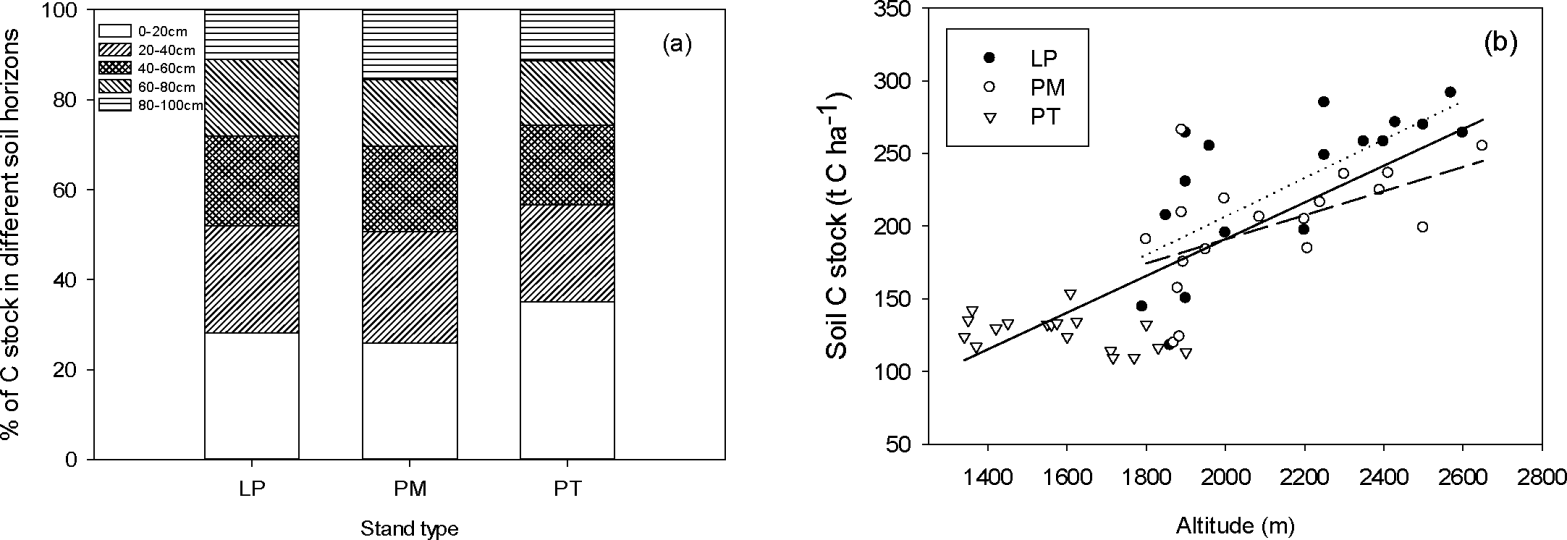
**Percentage of C stock in different soil horizons (a) and total soil C stock along the altitudinal gradient (b) in three coniferous forests on the Loess Plateau, China.** LP, *Larix principis-rupprechtii* forest; PM, *Picea meyerii* forest; PT, *Pinus tabulaeformis* forest. Solid, long dash and dotted lines represent significant relationships between soil C stock and altitude across the three forests (r^2^ = 0.61, p<0.001), within LP forest (r^2^ = 0.49, p = 0.001) and within PM forest (r^2^ = 0.29, p = 0.021) respectively in (b).

Soil C stocks down to 1m in depth in LP and PM forests were both significantly higher (228.1±12.1 and 200.4±9.3 t C ha^-1^) than that in PT forest (127.1±2.8 t C ha^-1^, Table 2A). Over 70% of the soil C was stored in the upper 60cm soil in all of these forests (Fig 4A). Total soil C stock significantly increased with altitude across the three forests and within LP and PM forests respectively (Fig 4B).

**Table 2:** C stock by ecosystem components and their proportions to total C stock in three coniferous forests on the Loess Plateau, China.

	LP	PM	PT	One-way ANOVA *p* values
a) Carbon density (t C ha^-1^)				
Vegetation	141.6(8.2)a	162.3(9.9)a	96.7(11.8)b	<0.001
Necromass	5.3(0.7)a	5.7(1.0)a	6.4(0.8)a	0.507
Soil	228.1(12.1)a	200.4(9.3)a	127.1(2.8)b	<0.001
Total	375.0(16.8)a	368.4(14.0)a	230.2(13.0)b	<0.001
b) Proportion in total C stock (%)				
Vegetation	38.0(1.7)a	44.0(1.8)a	40.0(2.3)a	0.109
Necromass	1.4(0.1)b	1.6(0.3)b	3.0(0.4)a	0.002
Soil	60.6(1.7)a	54.4(1.8)a	57.0(2.1)a	0.082

Note: Data are presented by plot means with 1 standard error in parentheses. There are 18 plots for each forest type. Different letters within same row indicate significant differences (p<0.05) among three forest types. LP, *Larix principis-rupprechtii* forest; PM, *Picea meyerii* forest; PT, *Pinus tabulaeformis* forest.

### Carbon density in total and ecosystem components

**Fig 5:**
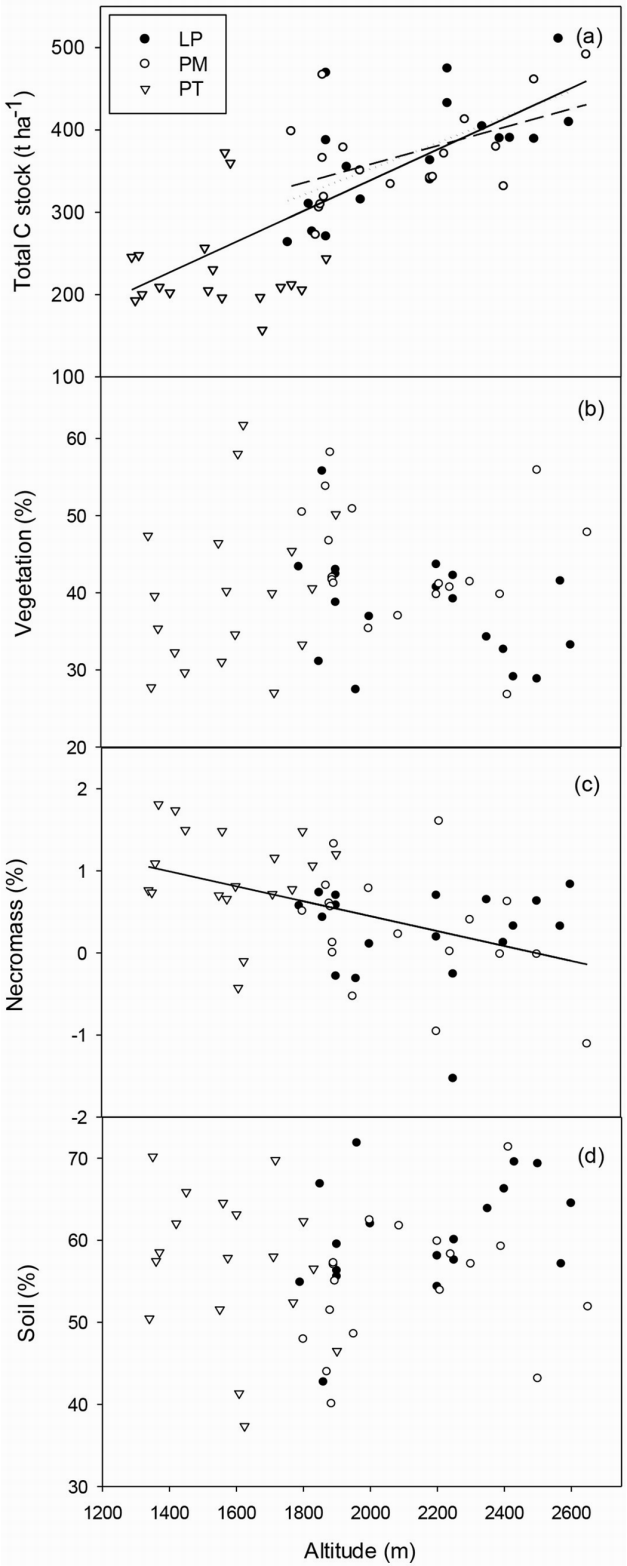
**Total C stock (a) and proportions of vegetation (b), necromass (c) and soil C stocks (d) to total C stock along the altitudinal gradient in three coniferous forests on the Loess Plateau, China.** LP, *Larix principis-rupprechtii* forest; PM, *Picea meyerii* forest; PT, *Pinus tabulaeformis* forest. The proportion of necromass to total C stock was log-transformed. Solid lines represent significant linear relationships between altitude and total C stock (r^2^ = 0.56, p<0.001) and the proportion of necromass to total C stock (r^2^ = 0.22, p<0.001) respectively across the three forests. Dotted and long dash lines represent significant linear relationships between altitude and total C stock within LP forest (r^2^ = 0.39, p = 0.006) and PM forest (r^2^ = 0.25, p = 0.033) respectively.

Both LP and PM forests had similar and significantly higher total C densities (375.0±16.8 and 368.4±14.0 t C ha^-1^respectively) than PT forest (230.2±13.0 t C ha^-1^, Table 2A). Similar to soil C stock, total C density significantly increased with altitude across the three forests and within LP and PM forests (Fig 5A).

More carbon was stored in soil than in vegetation in these three forests with no significant differences among forest types (Table 2A). Although accounting for only a small percentage of total C stock, the proportion of necromass C stock was higher in PT forest than in LP and PM forests, and also decreased with increasing altitude across forest types (Table 2B and Fig 5C). Neither the proportion of vegetation C stock nor the proportion of soil C stock changed with altitude across and within forest types (Fig 5B and 5D).

## Discussion

### Carbon stocks in three coniferous forests

C accumulation in forest ecosystems is generally increasing with stand development and thus a time-dependent process[33]. Climate and edaphic factors are also influential during this process. In our study, vegetation C stocks of three coniferous forests on the Loess Plateau were comparable to those of primary coniferous forests in temperate moist zone in northeast China ranging from 163 to 183 t C ha^-1^[22], but far higher than the provincial average (82 t C ha^-1^), the national average of evergreen coniferous forests (79 t C ha^-1^) and the average of temperate coniferous forests in northern hemisphere (62 t C ha^-1^) respectively[29, 35–36], despite they were at the mid-stage of stand development (around 65 yrs, Table 1A). This result showed the strong resilience of these secondary forests without further disturbances even under a less favorable semi-arid climate. However, vegetation C stocks of these forests were still less than that of old-growth forests both in quantity and in proportion to total C stock, such as those reported for old-growth fir forest (326 t C ha^-1^ and 51% respectively) in subalpine zone[33], indicating potential further C accumulation. In contrast, soil C pools in these forests were in greater proportion to total C stock and close to that of old-growth forests (256 t C ha^-1^) in subalpine zone and national average of evergreen coniferous forests (180 t C ha^-1^) in quantity[33, 35], which were also reported in secondary forests elsewhere in China[37–40]. High soil C density could also be partly due to well-developed soil C pools before disturbances in the fine-textured thick soil layer composed of aeolian loess without any clay content[41]. In total, these secondary forests had generally higher C stocks than local plantations (104 t C ha^-1^) converted from farmland[28], being an important C sink on Loess Plateau.

Among forest types, total C stocks of LP and PM forests were similar and significantly higher than that of PT forest because of both higher vegetation and soil C stocks. Higher and similar vegetation C stocks in LP and PM forests could not be explained by species-specific growth traits, because Prince Rupprecht’s larch and Chinese pine are fast-growth pioneers and Meyer spruce is mid-successional with relatively slow growth while these three forests had similar ages. A lower stand density in PT forest, due to a wider crown of Chinese pine than other two conifers, may lead to lower biomass increase measured by MAI. By utilizing the growing space left by overstory Chinese pines, the shrub layer in PT forest actually accumulated much higher biomass than in other two forests, being an unneglectable source to vegetation C stock of this forest type. Different vegetation C stocks of the three forests could also be explained by their different altitudinal distributions (Table 1A), since cooler temperature combined with higher precipitation at high altitudes is suggested being favorable to forest C accumulation[42].

Different soil C stocks among the three forests could be explained by their different altitudinal distributions, as vegetation and climate are key factors determining spatial distribution pattern of soil C storage in north China[43]. Higher vegetation biomass accumulated in LP and PM forests in the high altitudinal range could mean higher C input through litterfall and more importantly fine-root turnover, which is a major source of soil C input[44]. Soil C output is primarily determined by soil respiration[22]. Although whether soil respiration is affected by temperature is still in debate and depending on soil physical and chemical properties[45–47], the influence of precipitation on soil processes seems to be minimum[48]. Therefore, reduced activities of soil microorganisms and fauna at low temperature in the high altitudinal range of the study area were speculated to be favorable to soil C storage in LP and PM forests.

It was noted that although necromass C storage in the three forests were similar in our study, CWD and forest floor necromass showed opposite patterns. Although not significant, CWD necromass in LP and PM forests were generally higher than in PT forest, possibly due to two reasons. First, CWD was mainly composed of snags in our study with absence of fallen trees on the floor, which could be explained by relatively young ages of these forests, whereas less intense competition among individual trees in PT forest under a lower stand density might result fewer snags and thus a lower C stock of CWD. Second, low temperature in the high altitudinal range was unfavorable to CWD decomposition, thus may benefit C storage in CWD[49].

Forest floor necromass was highest in PT forest in the low altitudinal range, in contrary to the assumption of higher decomposition rates under high temperature[50]. Such discrepancy could not be fully explained by litterfall differences in amount, decomposition characteristics and stand development stage among forest types as suggested[51], because Prince Rupprecht’s larch is deciduous and Meyer spruce and Chinese pine are both evergreen. In the meantime, lower stand density in PT forest implied potentially less litter input from the canopy. Higher floor necromass in PT forest could be attributed to proliferation of shrubs in its understory and higher foliage turnover rates of shrub species than trees, but such contribution was limited since shrub layer biomass only accounted for less than 20% of vegetation biomass. More importantly, the climate in our study area is semi-arid and evapotranspiration usually exceeds precipitation, especially in the low altitudinal range[23]. In a meta-analysis, Liu et al.[52] found that precipitation plays a more important role than temperature in determination of litterfall amount on the floor in Eurasia forests under the arid climate. On the other hand, precipitation is also an important factor affecting litter decomposition[48, 53].Therefore, high floor necromass in PT forest could also be due to increased litterfall input from canopy and decreased litter decomposition rate both caused by low precipitation in the low altitudinal range of the study area, even a higher temperature in this range may benefit its decomposition. This result highlighted the confounding effect of moisture on forest litter C stock in the semi-arid zone.

### Carbon stocks along the altitudinal gradient

Changing climate conditions along altitudinal gradients can significantly affect plant growth and therefore their C accumulation in mountain ecosystems. Lower forest biomass at high altitudes were observed and assumed to be caused by cold temperature in temperate, sub-tropical and tropical forests[17, 22]. To the contrary, our study found a positive linear relationship between vegetation C stock and altitude across three forests. Such result may be explained by concurrent temperature and precipitation changes along the altitudinal gradient and the moderate altitudes in the study area. First, temperature and precipitation change with altitude synchronously but in opposite directions. Lower temperature with higher precipitation at high altitudes could create favorable water conditions for tree biomass accumulation and thus vegetation C stock, since water deficit is the limiting factor on tree growth on Loess Plateau and the majority of vegetation C stock in these forests was stored in the tree layer. A positive relationship between precipitation and aboveground biomass was suggested as the reason for forest biomass increase with altitude at Mt. Kilimanjaro in Africa[20]. Fehse et al.[17] also demonstrated high altitude secondary forests under favorable site conditions were not inferior in biomass accumulation and productivity compared with low altitude forests. Second, the three forests in our study are distributed on montane area, where the climate is less harsh than that in sub-alpine and alpine zones[32]. It is possible that the temperature at highest altitude of the sampled stands is still not too low to limit the growth of these tree species. In fact, two tree ring studies carried out in the study area and an adjacent area respectively showed that the radial growths of Prince Rupprecht’s larch and Meyer spruce were only limited by temperature over around 2600m a.s.l.[54–55]. Above this altitude, there is little land area left, mostly on up-slopes and mountain tops, thus only few stands were sampled in our study. In partial concordance with the results of these two studies, the mean annual increment (MAI) of vegetation biomass in LP forest was peaked at mid altitudes and decreased with increasing altitude thereafter, although MAIs of these three forests generally increased with altitude. Therefore, decreasing water deficit with increasing altitude and minor or no cold stress at high altitudes contributed to the increase of forest biomass with increasing altitude in the study area. However, such relationship was not significant within each forest type, probably because these forests were immature and still not peaked in biomass accumulation.

Biomass of the shrub and herb layers showed contrasting patterns along the altitudinal gradient across and within forest types in our study. In accordance with the results of similar studies[22], shrub layer biomass decreased with increasing altitude and such decrease was more prominent in LP and PM forests possibly due to the temperature decline along the altitudinal gradient while water conditions were generally improved in the understory comparing to that in the open irrespective of altitude[10]. Herb layer biomass increased with altitude in general, contradicting the general conclusion[21]. In the studies where understory herb biomass showed an increasing pattern with increasing altitude, relatively open canopy at high altitudes was inferred as the cause[20], which was not the case in our study in which all sampled stands were well stocked. Competition from understory shrubs and regenerated trees could explain the opposite trend of herb layer biomass along the altitudinal gradient comparing to that of shrub layer biomass in general, but not within each forest type. Therefore, it is likely that under the intense competition of shrubs in PT forest fairly unchanged with increasing altitude, alleviated surface soil water conditions in the upper part of the low altitudinal range caused the positive relationship between herb layer biomass and altitude.

Soil C pool size generally shows an increasing trend with increasing altitude[22]. Same trend was found across the three forests in our study and more pronounced in LP and PM forests distributed in the high altitudinal range. Significant linear relationships between soil C stock and altitude in LP and PM forests, driven by the effect of decreasing temperature on soil C output rather than higher C input from the vegetation, also accounted for significant increases of ecosystem C stock along the altitudinal gradient in these two forests. Invariable soil C stock along the altitudinal gradient in PT forest could be due to insensitivity of soil C stock of this forest type to temperature and precipitation under a semi-arid climate. For example, Gao et al.[56] found that soil C densities in natural Chinese pine stands at the development stages from near-mature to over-mature were not affected by soil water content. Sun et al.[57] also reported similar soil C stocks in near-mature Chinese pine plantations at different sites with contrasting mean annual temperatures and precipitations on Loess Plateau. However, since the altitudinal ranges in our study and the other two studies (from 1000-2000m a.s.l.) are relatively narrow for Chinese pine comparing to its wide distribution along the altitudinal gradient in central China (from 20-3000m a.s.l.), our conclusion should be further examined and confirmed.

C partitioning in forest ecosystems is highly variable because of different C turnover times and different responses of ecosystem components, i.e. vegetation, necromass and soil, to environmental factors[22, 51]. In the contrary, our study found that C allocations to vegetation and soil did not change with the altitude, because of similar responses of vegetation and soil C stocks to increasing altitude. Although an insignificant part of total C stock (less than 6%), necromass C allocation decreased with increasing altitude across forest types same as forest floor necromass (Fig 3B), due to both possible higher litterfall amount and lower litter decomposition rate on the floor in the low altitudinal range and an increasing total C stock with increasing altitude as explained.

### Implications to forest carbon balance on Loess Plateau under future climate change

The climate on Loess Plateau had been increasingly warm and dry in the past century evidenced by increasing temperature accompanied with decreasing precipitation[58–59]. Under such a climatic trend, the positive linear relationship between forest C stock and altitude in our study suggests that vegetation and soil C accumulation in montane forests on Loess Plateau could be further limited. Although MAI of LP forest showed a unimodal pattern along the altitudinal gradient, the effect of climate warming on tree growth would be less beneficial to further biomass accumulation of LP forest at the high altitudinal range because of small land area on mountain tops. Since LP and PM forests in the high altitudinal range sequestrated and stored more carbon in biomass than PT forest in the low altitudinal range, the predicated climate warming would not benefit biomass accumulation in these two forests by increasing growth temperature, but would slow such process by accompanied aggravation of water conditions in terms of less precipitation and higher evapotranspiration. The potential limited biomass accumulation in the study area was in contrast to the prediction in temperate moist zone[60–61]. The influence of water on forest growth on Loess Plateau would be even greater under the future climate, considering the uneven temporal distribution of annual precipitation, of which about two third is in July and August as rainfall[23]. Drier climate in the future would also cause more litterfall and lower litter decomposition rate, resulting more floor necromass as seen in PT forest. The increasing trend of soil C pool along the altitudinal gradient in LP and PM forests suggests the potential soil C loss in these two forests under the future climate. Similar results were also predicted in temperate moist forests in northwest China[60–61]. Overall, it is highly likable that climate warming would negatively impact the carbon balance in these three forests, changing them from current C sinks into C sources, especially for LP and PM forests in which vegetation biomass was affected more by precipitation than temperature and soil C stock was sensitive to temperature increase.

Afforestation and reforestation of montane area are key to the ecological restoration on Loess Plateau. In the past, Prince Rupprecht’s larch has been widely used in most artificial plantations because of its fast-growth trait. In light of the limiting effect of water conditions on biomass accumulation of three coniferous forests found in our study, local forest managers should consider broadleaved species instead, such as birches and oaks, to sequestrate more carbon under a warmer climate, because deep-rooted broadleaves are expected to perform better than shallow-rooted conifers under water stress and grow faster at a higher temperature. Introduction of seedlings of broadleaves or breeding of those naturally regenerated in the understory of these forests could not only help to keep a positive carbon balance in these forests under the future climate, but also help to increase their species and structural diversities for better ecological services[25, 62]. Meanwhile, decreasing shrub layer biomass with increasing altitude in LP and PM forests implies the invasion of shrub species into the forest understory under a warmer climate, being a potential threat to forest regeneration. In future forest management practices, understory shrub species should be monitored and promptly eliminated to reduce their competition for water under probably aggravated water conditions caused by climate warming.

## Conclusions

The three coniferous secondary forests had high total C stocks driven by both high vegetation biomass and high soil C stocks even at a mid-development stage. Among forest types, vegetation and soil C stocks were higher in LP and PM forests than in PT forest, while an inverse pattern was observed for forest floor necromass. Understory shrubs accounted for about 20% of total biomass in PT forest probably due to the high temperature in the low altitudinal range and much growing space left by widely spaced Chinese pines. Vegetation and soil C stocks both increased with altitude across the three forests, causing fairly unchanged C partitioning among ecosystem components along the altitudinal gradient, but were driven by different factors. The increase of vegetation C stock could be explained by the effect of increasing precipitation along the altitudinal gradient on tree growth with minor or no temperature limitation in the high altitudinal range. The increase of soil C stock was the result of decreasing temperature with altitude. Furthermore, forest floor necromass decreased with increasing altitude due to the confounding effect of water conditions on litterfall amount and decomposition. Our results suggest that although higher MAIs of LP and PM forests indicated higher potential in C accumulation, these two forests would suffer more losses in C storage under climate warming since their C sequestrations were mainly influenced by water conditions and their soil C pools were temperature limited.

## Supporting information

S1 Table Relationship between diameter at breast height (DBH, cm) and tree height (H, m) by species. Note: Coefficients are estimated using model (1). All models are significant at p<0.001.
(DOCX)

S2 Table Relationship between diameter at breast height (DBH, cm) and tree age (yrs) by species. Note: Coefficients are estimated using model (2). All models are significant at p<0.001.
(DOCX)

S3 Table Biomass models for trees with a height >1.3m by species. Note: AGB, aboveground biomass; BGB, belowground biomass; Stem, stem biomass; D, diameter at 1.3m; H, height. Biomass model of Prince Rupprecht’s larch was developed by Zhang and Shangguan [27]; models of Chinese pine and Cathay poplar were developed by Wang et al. [28]; model of East-liaoning oak was developed by Wang et al. [29]; models of Asian white birch and Meyer spruce were compiled by State Forestry Administration [30].
(DOCX)

S4 Table Biomass models for trees with a height <1.3m by species. Note: All models are significant at p<0.001. RCD, root collar diameter; H, height.
(DOCX)

S1 File Note: Stand characteristics, biomass and total carbon stock by components in selected stands. Abbreviations: BA, basal area; DomPer, percentage of basal area of dominated species; TB, total biomass; SB, shrub layer biomass; HB, herb layer biomass; MAI, mean annual increment; CWD, necromass of coarse wood debris; Floor, floor necromass; TC, total C stock; NC, necromass C stock; TSC, total soil C stock; 20SC-100SC, soil C stocks in 0-20cm, 20-40cm, 40-60cm, 60-80cm and 80-100cm soil layers respectively. SI units are used for all variables, i.e. meter for elevation, stems per hectare for stand density (DBH≥10 cm), square meters per hectare for BA, ton per hectare per year for MAI, ton per hectare for total biomass by components and ton C per hectare for total C stock by components.
(CSV)

## References

[1] Körner C, Ohsawa M. Mountain systems. In Ecosystems and human well-being: findings of the Condition and Trends Working Group v.1: current state and trends, Hassan R.M, Scholes U.R, Ash N, Eds, Island Press: Washington, USA, 2005; pp. 683–713. ISBN: 9781559632287

[2] Fang J, Chen A, Peng C, Zhao S, Ci L. Changes in forest biomass carbon storage in China between 1949 and 1998. Science 2001; 292(5525), 2320–2322. doi: 10.1126/science.1058629 11423660 10.1126/science.1058629

[3] Zhang J, Li B. The characteristics of Forest landscape in the Loess Plateau, China. J Mountain Sci 2006; 24(1):1–6. doi: 10.16089/j.cnki.1008-2786.2006.01.001

[4] Liao C, Luo Y, Fang C, Li B. Ecosystem carbon stock influenced by plantation practice: implications forest planting forests as a measure of climate change mitigation. PloS one 2010; 5(5), e10867 doi: 10.1371/journal.pone.0010867 20523733 10.1371/journal.pone.0010867 PMC2877715

[5] van Breugel M, Ransijn J, Craven D, Bongers F, Hall JS. Estimating carbon stock in secondary forests: decisions and uncertainties associated with allometric biomass models. For Ecol Manage 2011; 262(8), 1648–1657. doi: 10.1016/j.foreco.2011.07.018

[6] Wang N, Wang BT, Wang RJ, Cao XY, Wang WJ, Chi L. Study on Soil Carbon Density under Some Main Forest Types in the Central Part of Shanxi Province. Chin J Soil Sci 2013; 44(4):858–862. doi: 10.19336/j.cnki.trtb.2013.04.014

[7] Wang Y, Wang MB, Zhu SZ, Zhao TL. Carbon densities of major tree species in forests in southern Lüliang Mountains of Shanxi province, China. Chin J Ecol 2015; 34(2), 333–340. doi: 10.13292/j.1000-4890.2015.0046

[8] Yang XM, Cheng JM, Meng L, Han JJ. Carbon storage and density of forests in Ziwuling area of Loess Plateau. J Soil Water Conserv 2010; 24(6), 123–125. doi: 10.13870/j.cnki.stbcxb.2010.06.051

[9] Meng L, Cheng JM, Yang XM, Han JJ, Fan WJ, Hu XJ. Artificial *Pinus tabulaeformis* carbon storage and density in Ziwuling Forest area on the loess plateau. Bull Soil Water Conserv 2010; 30 (2), 133–137. doi: 10.13961/j.cnki.stbctb.2010.02.048

[10] Liu N, Wang T, Feng Q, Guo J, Zhang Y, Wang J. Transplantation of *Larix principis-rupprechtii* Mayr. and *Picea meyeri* Rehd. seedlings to low altitude and two contrasting light environments reveals climate warming effects on early seedling performance. Scand J Forest Res 2016; 31, 46–55. doi: 10.1080/02827581.2015.1055790

[11] Zhang X, Wang M, Liang X. Quantitative classification and carbon density of the Forest vegetation in Lüliang Mountains of China. Plant Ecol 2009; 1, 1–9. doi: 10.1007/s11258-008-9507-x

[12] Körner C. The use of ‘altitude’ in ecological research. Trends Ecol Evol 2007; 22:569–574. doi: 10.1016/j.tree.2007.09.006 17988759 10.1016/j.tree.2007.09.006

[13] Homeier J, Breckle SW, Günter S, Rollenbeck RT, Leuschner C. Tree diversity, Forest structure and productivity along altitudinal and topographical gradients in a species‐rich Ecuadorian montane rain forest. Biotropica 2010;42(2), 140–148. doi: 10.1111/j.1744-7429.2009.00547.x

[14] Reinhardt K, Castanha C, Germino MJ, Kueppers LM. Ecophysiological variation in two provenances of *Pinus flexilis* seedlings across an elevation gradient from Forest to alpine. Tree Physiol 2011; 31(6), 615–625. doi: 10.1093/treephys/tpr055 21757486 10.1093/treephys/tpr055

[15] Shi P, Körner C, Hoch G. A test of the growth‐limitation theory Forest alpine tree line formation in evergreen and deciduous taxa of the eastern Himalayas. Funct Ecol 2008; 22(2), 213–220. doi: 10.1111/j.1365-2435.2007.01370.x

[16] Smith WK, Germino MJ, Johnson DM, Reinhardt K. The altitude of alpine treeline: a bellwether of climate chang effects. Bot Rev 2009; 75(2), 163–190.

[17] Fehse J, Hofstede R, Aguirre N, Paladines C, Kooijman A, Sevink J. High altitude tropical secondary forests: a competitive carbon sink? For Ecol Manage 2002; 163(1), 9–25. doi: 10.1016/S0378-1127(01)00535-7

[18] Garkoti SC. Estimates of biomass and primary productivity in a high-altitude maple Forest of the west central Himalayas. Ecol Res 2008; 23(1), 41–49. doi: 10.1007/s11284-007-0355-2

[19] Sheikh MA, Kumar M, Bussmann RW. Altitudinal variation in soil organic carbon stock in coniferous subtropical and broadleaf temperate forests in Garhwal Himalaya. Carbon Balance Manage 2009; 4(1), 6 doi: 10.1186/1750-0680-4-6 19706175 10.1186/1750-0680-4-6 PMC2745406

[20] Ensslin A, Rutten G, Pommer U, Zimmerman R, Hemp A, Fischer M. Effects of elevation and land use on the biomass of trees, shrubs and herbs at Mount Kilimanjaro. Ecosphere 2015; 6(3):1–15. doi: 10.1890/ES14-00492.1

[21] Kharkwal G, Mehrotra P, Rawat YS. Biomass production of herb species in broad leaf forests in Kumaun Himalaya, India. J For Res 2010; 21(3), 355–360. doi: 10.1007/s11676-010-0067-2

[22] Zhu B, Wang X, Fang J, Piao S, Shen H, Zhao S, et al Altitudinal changes in carbon storage of temperate forests on Mt Changbai, Northeast China. J Plant Res 2010; 123(4), 439–452. doi: 10.1007/s10265-009-0301-1 20127501 10.1007/s10265-009-0301-1

[23] Xiao Y, Tian SB, Li YP, Zhang YX, Guo JP. A study on the relationship between the local climatic-geographic gradient and the bio-climatic indices in Guandi Mountains. J of Shanxi Agricultural University (Nat Sci) 1998; 18:5–9. doi: 10.13842/j.cnki.issn1671-8151.1998.01.002

[24] Tang JL, Liu YL, Li XB, Duan YG. Analysis of plant communities and environmental gradient on Guandi moutain. J Beijing Forestry University 1995; 17:36–43. doi: 10.13332/j.1000-1522.1995.04.006

[25] Liu N, Wang H, Nan H. Structural diversity closely associated with canopy species diversity and stand age in species-poor montane forests on Loess Plateau of China. Pol J Ecol 2017; 65(2), 183–193. doi: 10.3161/15052249PJE2017.65.2.002

[26] Wang C. Biomass allometric equations Forest 10 co-occurring tree species in Chinese temperate forests. For Ecol Manage 2006; 222(1), 9–16. doi: 10.1016/j.foreco.2005.10.074

[27] Zhang J, Shangguan Y. On synecological features and biomass of *Larix principis-rupprechtii* Forest in Guandi mountain, Shanxi Province. J Shanxi University (Nat Sci) 1993; 15(1):72–77. doi: 10.13451/j.cnki.shanxi.univ(nat.sci.;1992.01.016

[28] Wang N, Wang BT, Wang RJ, Cao XY, Wang WJ, Chi L. Biomass allocation patterns and allometric models of *Populus davidiana* and *Pinus tabulaeformis* Carr. in west of Shanxi Province. Bull Soil Water Conser 2013b; 33(2), 151–155. doi: 10.13961/j.cnki.stbctb.2013.02.061

[29] Wang N. Study on distribution patterns of carbon density and carbon stock in the Forest ecosystem of Shanxi Province. Doctoral Dissertation, Beijing Forestry University, China, 2014. Available from: http://cdmd.cnki.com.cn/Article/CDMD-10022-1014324783.htm.

[30] State Forestry Administration. Guidelines on carbon accounting and monitoring Forest afforestation project Chinese Forestry Press: Beijing, China, 2014; pp. 55–65. ISBN: 9787503873676

[31] Ma Q, Chen X, Wang J, Lan C, Kang F, Cao W, et al Carbon content rate in constructive species of main Forest types in northern China. J Beijing Forestry University 2002;24, 96–100. doi: 10.13332/j.1000-1522.2002.z1.020

[32] Chen X, Ma Q, Kang F, Cao W, Zhang G, Chen Z. Studies on the biomass and productivity of typical shrubs in Taiyue mountain, Shanxi province. For Res 2002; 15(3), 304–309. doi: 10.17521/cjpe.2016.0131

[33] Zhang Y, Gu F, Liu S, Liu Y, Li C. Variations of carbon stock with Forest types in subalpine region of southwestern China. For Ecol Manage 2013; 300, 88–95. doi: 10.1016/j.foreco.2012.06.010

[34] R Core Team (2016). R: A language and environment for statistical computing R Foundation for Statistical Computing, Vienna, Austria URL https://www.R-project.org/.

[35] Li KR, Wang SQ, Cao MK. Vegetation and soil carbon storage in China. Sci China Ser D Earth Sci 2004; 47, 49–57. doi: 10.1360/02yd0029

[36] Thurner M, Beer C, Santoro M, Carvalhais N, Wutzler T, Schepaschenko D, et al Carbon stock and density of northern boreal and temperate forests. Glob Ecol Biol 2014; 23(3), 297–310. doi: 10.1111/geb.12125

[37] Fang J, Liu G, Zhu B, Wang X, Liu S. Carbon budgets of three temperate Forest ecosystems in Dongling Mt, Beijing, China. Sci China Ser D, Earth Sci 2007; 50(1), 92–101. doi: 10.1007/s11430-007-2031-3

[38] Guo LB, Gifford RM. Soil carbon stocks and land use change: a meta analysis. Glob Chang Biol 2002; 8(4), 345–360. doi: 10.1046/j.1354-1013.2002.00486.x

[39] Lal R. Soil carbon sequestration in China through agricultural intensification, and restoration of degraded and desertified ecosystems. Land Degrad Dev 2002; 13(6), 469–478. doi: 10.1002/ldr.531

[40] Zhao J, Kang F, Wang L, Yu X, Zhao W, Song X, et al Patterns of biomass and carbon distribution across a chronosequence of Chinese pine (*Pinus tabulaeformis*) forests. PLoS one, 2014; 9(4), e94966 doi: 10.1371/journal.pone.0094966 24736660 10.1371/journal.pone.0094966 PMC3988105

[41] Yang Y, Li P, Ding J, Zhao X, Ma W, Ji C, Fang J. Increased topsoil carbon stock across China's forests. Glob Chang Biol 2014; 20: 2687–2696. doi: 10.1111/gcb.12536 24453073 10.1111/gcb.12536

[42] Keith H, Mackey BG, Lindenmayer DB. Re-evaluation of Forest biomass carbon stocks and lessons from the world’s most carbon-dense forests. Proc Natl Acad Sci USA 2009; 106:11635–11640. doi: 10.1073/pnas.0901970106 19553199 10.1073/pnas.0901970106 PMC2701447

[43] Yang YH, Mohammat A, Feng JM, Zhou R, Fang JY. Storage, patterns and environmental controls of soil organic carbon in China. Biogeochemistry 2007; 84, 131–141. doi: 10.1007/s10533-007-9109-z

[44] Yuan ZY, Chen HYH. Fine root dynamics with stand development in the boreal forest[J]. Functional Ecology, 2012, 26(4):991–998. doi: 10.1111/j.1365-2435.2012.02007.x

[45] Conant RT, Steinweg JM, Haddix ML, Paul EA, Plante AF, Six J. Experimental warming shows that decomposition temperature sensitivity increases with soil organic matter recalcitrance. Ecology 2008; 89(9), 2384–2391. doi: 10.1890/08-0137.1 18831158 10.1890/08-0137.1

[46] Conant RT, Ryan MG, Ågren GI, Birge HE, Davidson EA, Eliasson PE, et al Temperature and soil organic matter decomposition rates–synthesis of current knowledge and a way forward. Glob Chang Biol 2011; 17(11), 3392–3404. doi: 10.1111/j.1365-2486.2011.02496.x

[47] Giardina CP, Ryan MG. Evidence that decomposition rates of organic carbon in mineral soil do not vary with temperature. Nature 2000; 404(6780), 858–861. doi: 10.1038/35009076 10786789 10.1038/35009076

[48] Salamanca EF, Kaneko N, Katagiri S. Rainfall manipulation effects on litter decomposition and the microbial biomass of the Forest floor. Appl Soil Ecol 2003; 22(3), 271–281. doi: 10.1016/S0929-1393(02)00153-1

[49] Herrmann S, Bauhus J. Effects of moisture, temperature and decomposition stage on respirational carbon loss from coarse woody debris (CWD) of important European tree species. Scand J For Res 2013; 28(4), 346–357. doi: 10.1080/02827581.2012.747622

[50] Reichstein M, Subke JA, Angeli AC, Tenhunen JD. Does the temperature sensitivity of decomposition of soil organic matter depend upon water content, soil horizon, or incubation time? Glob Chang Biol 2005; 11(10), 1754–1767. doi: 10.1111/j.1365-2486.2005.001010.x

[51] Pregitzer KS, Euskirchen ES. Carbon cycling and storage in world forests, biome patterns related to Forest age. Glob Chang Biol 2004; 10, 2052–2077. doi: 10.1111/j.1365-2486.2004.00866.x

[52] Liu C, Westman CJ, Björn B, Werner K, Wang Gary Z; Man R. Variation in litterfall-climate relationships between coniferous and broadleaf forests in Eurasia. Glob Ecol Bio 2010; 13(2), 105–114. doi: 10.1111/j.1466-882X.2004.00072.x

[53] Wang Y, Wang ZL, Wang H, Guo C, Bao W. Rainfall pulse primarily drives litterfall respiration and its contribution to soil respiration in a young exotic pine plantation in subtropical China. Can J For Res 2012; 42(4), 657–666. doi: 10.1139/x2012-017

[54] Cai Q, Liu Y. Climatic response of three tree species growing at different elevations in the Lüliang Mountains of Northern China. Dendrochronologia 2013; 31(4), 311–317. doi: 10.1016/j.dendro.2012.07.003

[55] Zhang WT, Jiang Y, Dong MY, Kang MY, Yang HC. Relationship between the radial growth of *Picea meyeri* and climate along elevations of the Luyashan Mountain in North-Central China. For Ecol Manage 2012; 265, 142–149. doi: 10.1016/j.foreco.2011.10.017

[56] Gao J, Guo ZJ, Liu YH. Soil organic carbon distribution and its influencing factors of Beijing Songshan natural Chinese pine forests. Chin J Ecol 2016; 35(10), 2707–2713. doi: 10.13292/j.1000-4890.201610.032

[57] Sun M, Guan J, Wu C, Yue J, Li G, Du S. Carbon storage features of *Pinus tabulaeformis* plantations that are approaching maturity at three sites across a precipitation gradient in western loess plateau. Acta Ecologica Sinica 2017; 37(8), 2665–2672. doi: 10.5846/stxb201512262562

[58] Zhang LH, Yan JP, Liu LS. Climate change and drought and flood disasters trend in Shanxi. J Arid Land Resour Environ 2013; 27(5):120–125. doi: 10.13448/j.cnki.jalre.2013.05.027

[59] Zhou XH, Zhao JB. Climatic Change and Vegetation Restoration on the Loess Plateau. Arid Zone Res 2005; 22(1):116–119.

[60] Peng CH, Zhou XL, Zhao SQ, Wang XP, Zhu B, Piao SL, et al Quantifying the response of Forest carbon balance to future climate Chang in Northeastern China: Model validation and prediction. Glob Planet Chang 2009; 69, 179–194. doi: 10.1016/j.gloplacha.2008.12.001

[61] Xie Z, Zhu J, Liu G, Cadisch G, Hasegawa T, Chen C, et al Soil organic carbon stocks in China and changes from 1980s to 2000s. Glob Chang Biol 2007; 13(9), 1989–2007. doi: 10.1111/j.1365-2486.2007.01409.x

[62] Liu B, Liu Q, Daryanto S, Guo S, Huang Z, Wang Z, et al Responses of Chinese fir and *Schima superba* seedlings to light gradients: Implications for the restoration of mixed broadleaf-conifer forests from Chinese fir monocultures. For Ecol Manage 2018; 419–420, 51–57. doi: 10.1016/j.foreco.2018.03.033

