# Supplementary material for: Carbon stocks of three secondary coniferous forests along an altitudinal gradient on Loess Plateau in inland China
Source: PLoS One. 2018 May 3;13(5):e0196927. doi: 10.1371/journal.pone.0196927 (PMC5933742; doi:10.1371/journal.pone.0196927)
Supplement: S1 Table — Note: Coefficients are estimated using model (1). All models are significant at p<0.001. (DOCX) [file pone.0196927.s001.docx]

S1 Table. Relationship between diameter at breast height (DBH, cm) and tree height (H, m) by species.

| Species | Coefficients | | | r2 | No. of trees | H range | DBH range |
| --- | --- | --- | --- | --- | --- | --- | --- |
|  | a | b | c |  |  |  |  |
| Prince Rupprecht’s larch | 42.832 | 0.015 | 1.061 | 0.971 | 30 | 1.5-22.0 | 0.5-42.0 |
| Meyer spruce | 44.1396 | 0.016 | 1.214 | 0.958 | 29 | 1.8-21.1 | 0.9-49.8 |
| Chinese pine | 1143.379 | 0.001 | 1.077 | 0.988 | 24 | 1.4-17.2 | 0.7-42.1 |
| East-liaoning oak | 170.356 | 0.001 | 0.580 | 0.870 | 27 | 3.4-12.0 | 2.8-31.0 |
| Asian white birch | 1579.081 | 0.001 | 0.860 | 0.906 | 26 | 3.2-19.9 | 1.7-42.2 |

Note: Coefficients are estimated using model (1). All models are significant at p<0.001.
