# Supplementary material for: Carbon stocks of three secondary coniferous forests along an altitudinal gradient on Loess Plateau in inland China
Source: PLoS One. 2018 May 3;13(5):e0196927. doi: 10.1371/journal.pone.0196927 (PMC5933742; doi:10.1371/journal.pone.0196927)
Supplement: S2 Table — Note: Coefficients are estimated using model (2). All models are significant at p<0.001. (DOCX) [file pone.0196927.s002.docx]

S2 Table. Relationship between diameter at breast height (DBH, cm) and tree age (yrs) by species.

| Species | Coefficients | | | r2 | No. of trees | Age range | DBH range |
| --- | --- | --- | --- | --- | --- | --- | --- |
|  | a | b | c |  |  |  |  |
| Prince Rupprecht’s larch | 6 | 2.861 | 0.983 | 0.753 | 54 | 10-92 | 7.7-47.0 |
| Meyer spruce | 11 | 0.990 | 1.181 | 0.904 | 30 | 13-89 | 0.9-49.8 |
| Chinese pine | 7 | 0.001 | 1.077 | 0.988 | 38 | 9-77 | 4.5-45 |

Note: Coefficients are estimated using model (2). All models are significant at p<0.001.
