# Supplementary material for: Carbon stocks of three secondary coniferous forests along an altitudinal gradient on Loess Plateau in inland China
Source: PLoS One. 2018 May 3;13(5):e0196927. doi: 10.1371/journal.pone.0196927 (PMC5933742; doi:10.1371/journal.pone.0196927)
Supplement: S3 Table — Note: AGB, aboveground biomass; BGB, belowground biomass; Stem, stem biomass; D, diameter at 1.3m; H, height. Biomass model of Prince Rupprecht’s larch was developed by Zhang and Shangguan [27]; models of Chinese pine and Cathay poplar were developed by Wang et al. [28]; model of East-liaoning oak was developed by Wang et al. [29]; models of Asian white birch and Meyer spruce were compiled by State Forestry Administration [30]. (DOCX) [file pone.0196927.s003.docx]

S3 Table. Biomass models for trees with a height >1.3m by species.

| Species |  |  |
| --- | --- | --- |
| Prince Rupprecht’s larch | AGB | w=((D^2^H/10000)/(0.173+0.0383*(D^2^H/10000)))+(0.6744+30.1576*(D^2^H/10000))  +(188.1264* (D^2^H/10000)^1.0255^))+(50.1578*POWER(D^2^H/10000)^0.7871^) |
|  | BGB | w=50.1578* (D^2^H/10000)^0.7871^ |
|  | Stem | w=188.1264* (D^2^H/10000)^1.0255^ |
| Meyer spruce | AGB | w=0.09711* D^2.4198^ |
|  | BGB | w=0.02621* D^2.5266^ |
|  | Stem | w=0.0478* (D^2^H)^0.8665^ |
| Chinese pine | AGB | w=5.991+0.019*D^2^H |
|  | BGB | w=7.559+0.024*D^2^H |
|  | Stem | w=-1.3557+ (D^2^H)^0.86795^ |
| East-liaoning oak | AGB | w=0.126* (D^2^H)^0.838^ |
|  | BGB | w=0.0493* (D^2^H)^0.8514^ |
|  | Stem | w=0.004917* D^3.09503^ |
| Asian white birch | AGB | w=0.00016* (D^2^H)^1.1688^+0.00063* (D^2^H)^1.2781^+0.0319* (D^2^H)^0.93568^ |
|  | BGB | w=0.02275* (D^2^H)^0.91035^ |
|  | Stem | w=0.00063* (D^2^H)^1.2781^ |

Note: AGB, aboveground biomass; BGB, belowground biomass; Stem, stem biomass; D, diameter at 1.3m; H, height. Biomass model of Prince Rupprecht’s larch was developed by Zhang and Shangguan^[27]^; models of Chinese pine and Cathay poplar were developed by Wang et al. ^[28]^; model of East-liaoning oak was developed by Wang et al. ^[29]^; models of Asian white birch and Meyer spruce were compiled by State Forestry Administration^[30]^.
