# Supplementary material for: Carbon stocks of three secondary coniferous forests along an altitudinal gradient on Loess Plateau in inland China
Source: PLoS One. 2018 May 3;13(5):e0196927. doi: 10.1371/journal.pone.0196927 (PMC5933742; doi:10.1371/journal.pone.0196927)
Supplement: S4 Table — Note: All models are significant at p<0.001. RCD, root collar diameter; H, height. (DOCX) [file pone.0196927.s004.docx]

S4 Table. Biomass models for trees with a height <1.3m by species.

| Species | Aboveground biomass | r2 | No. of trees | Height range (m) | RCD range (cm) |
| --- | --- | --- | --- | --- | --- |
| Prince Rupprecht’s larch | w=0.121+0.017*RCD^2^H | 0.925 | 15 | 0.6-3.8 | 0.9-6.2 |
| Meyer spruce | w=-0.490+0.047*RCD^2^H | 0.940 | 13 | 0.5-2.6 | 0.8-8.4 |
| Chinese pine | w=0.419+0.012*RCD^2^H | 0.843 | 12 | 0.9-3.5 | 1.8-7.7 |
| East-liaoning oak | w=0.004+0.014*RCD^2^H | 0.788 | 10 | 0.2-2.1 | 0.6-5.5 |
| Asian white birch | w=-0.031+0.019*RCD^2^H | 0.990 | 15 | 0.9-4.2 | 1.0-6.0 |

Note: All models are significant at p<0.001. RCD, root collar diameter; H, height.
